# The association between overweight and prevalence of food allergy in Japanese children: a cross-sectional study

**DOI:** 10.1186/s12199-021-00960-2

**Published:** 2021-04-05

**Authors:** Koichiro Hayashi, Hiromasa Tsujiguchi, Daisuke Hori, Yohei Yamada, Yukari Shimizu, Thao Thi Thu Nguyen, Yuri Hibino, Yasuhiro Kambayashi, Akinori Hara, Hiroyuki Nakamura

**Affiliations:** 1 Department of Environmental and Preventive Medicine, Graduate School of Medical Sciences, Kanazawa University, Kanazawa, Japan; 2 Faculty of Medicine, University of Tsukuba, Tsukuba, Japan; 3 Department of Nursing, Faculty of Health Sciences, Komatsu University, Komatsu, Japan; 4 Department of Public Health, Faculty of Veterinary Medicine, Okayama University of Science, Imabari, Japan; 5 Advanced Preventive Medical Center, Kanazawa University, Kanazawa, Japan

**Keywords:** Asthma, Children, Food allergy, Gender, Overweight

## Abstract

**Background:**

Food allergy (FA) is a common disease in children, and its prevalence has increased in developed countries. The impact of overweight on children health also becomes an important social problem. However, the relationship between overweight and FA is still unclear. We examined the association between overweight and the prevalence of FA among Japanese children.

**Methods:**

We analyzed data obtained using a self-administered questionnaire from 1772 Japanese children. Weight groups according to body mass index cutoff points proposed by the International Obesity Task Force were used to create two groups: overweight and non-overweight. Children were separated into four age groups (3–6 years, 6–9 years, 9–12 years, and 12–15 years) to examine age differences. We performed univariate and multivariate logistic models to examine the association between overweight and FA.

**Results:**

The prevalence of FA was significantly higher in boys (10.6%, *p* = 0.014) than girls (4.5%) and girls (7.9%, *p* = 0.012) than boys (2.5%) for 6–9 and 12–15 age groups, respectively. While the prevalence of FA was significantly higher in overweight than non-overweight girls (26.1%, *p* = 0.005) in the 12–15 age group, no significant difference was found in boys. In girls, overweight was significantly associated with FA after adjustment for age and asthma (odds ratio 1.99, 95% confidence interval 1.01–3.89, *p* = 0.046).

**Conclusions:**

Our results showed that being overweight was significantly associated with a higher prevalence of FA in girls, but not in boys. Further prospective studies are necessary to find the causal relationship between overweight and FA.

## Background

Food allergy (FA) is a common disease in childhood and its prevalence has increased in developed countries [1, 2]. However, its etiology is unknown. Improved hygiene has been considered as a risk factor for FA due to decreased exposure to microorganisms that can induce allergic diseases (hygiene hypothesis) [3]. Decrease in the prevalence of food allergy is associated with increase in number of children [4]. Increase in the prevalence of food allergy is related to the history of skin infection or eczema [4]. The nationwide Japanese FA survey was previously conducted [5]. The prevalence of food allergy is 16.7%, 9.8%, 5.2%, and 4.0% in 3-year-old children, 0–6-year-old children, 0–5-year-old children, and 0–6-year-old children, respectively. The prevalence of food allergy from elementary school children to high school children is 2.5–4.45% [5].

Overweight (including obesity) has increased in Japan in recent years [6]. Overweight is an important issue in allergic diseases because a relationship between overweight and allergic diseases, particularly asthma, has been suggested [7–9]. The prevalence of obesity between 1996 and 2000 is 9.7%, 15.0%, and 8.5% in 6–8-year-old boys, 9–11-year-old boys, and 12–14-year-old boys in the National Nutrition Survey in Japan [5]. The prevalence of obesity between 1996 and 2000 is 10.3%, 12.2%, and 8.4% in 6–8-year-old girls, 9–11-year-old girls, and 12–14-year-old girls [5].

Children with asthma are likely to develop other allergic diseases, including FA, and FA is associated with more severe asthma [10]. However, the relationship between overweight and FA is not clear. Most studies examining the relationship between the growth of children and FA showed that FA is a risk factor for impaired growth because of the diet associated with its elimination in the case of FA [11–13], whereas there were few epidemiologic studies to examine the involvement of overweight in FA. Therefore, this study was conducted to examine the association between overweight and the prevalence of FA among Japanese children.

## Methods

### Study design and setting

Our cross-sectional, questionnaire-based survey was conducted in the town of Shika, Japan. Shika is a rural town located in Ishikawa prefecture in Japan and has a total population of almost 22,500. The Shika study (cohort study) has been conducted in Shika town since 2011. Children from 3 to 6 years old of 7 preschools (6 public nursery schools and 1 private kindergarten), 8 public elementary schools, and 2 public junior high schools in the town participated in this study in October–November 2013.

### Study population

We collected data from children in preschool (3–5 years old), elementary school (6–12 years old), and junior high school (12–15 years old). Education from elementary school to junior high school is compulsory in Japan; therefore, there is no home schooling in the area; thus, almost all children aged 3–15 years who lived in Shika participated in the present study. The teachers of each class distributed self-administered questionnaires to a total of 1,884 children. One thousand eight hundred and twenty-five sealed envelopes were collected and sent back to us without opening. Children with missing data on key variables (gender, age, height, weight, FA, and asthma) were excluded from the analyses (*n* = 53), leaving a total sample of 1772 children (878 boys and 894 girls) and a valid response rate of 94.1% (= 1772/1884).

### Questionnaire

The questionnaire comprised questions about gender, age, weight, height, and allergic diseases included in the International Study of Asthma and Allergies in Childhood (ISAAC) (spelled in Japanese) [14]. Questions in the ISAAC were completed at home either by children (children from junior high school) or by parents (children at sixth grade or lower). All other questions, including the FA, were completed by parents/guardians. FA was defined as answering “yes” to the question “Does your child have any food allergies?”. Asthma was defined as an answer of “yes” to the question “have you (or your child) ever been diagnosed with asthma?”.

### Definition of weight groups and age groups

Children’s weight and height were obtained from the questionnaire. Body mass index (BMI) was calculated as body weight in kilograms divided by height in meters squared (kg/m^2^). International age- and sex-specific cutoff points for BMI proposed by the International Obesity Task Force were used to define underweight, normal weight, overweight, and obesity [15, 16]. These cutoff points are linked to the widely accepted adult cutoff points of 18.5 kg/m^2^ (underweight), 25 kg/m^2^ (overweight), and 30 kg/m^2^ (obese). We divided these four groups into two groups of overweight (overweight and obese) and non-overweight (underweight and normal weight) to investigate the relationship between overweight and FA.

Age groups were classified into four categories: 3–6 years (preschool), 6–9 years (first–third grades of elementary school), 9–12 years (fourth–sixth grades of elementary school), and 12–15 years (junior high school) to examine age differences.

### Statistical analysis

The chi-square test and Fisher’s exact test were used for categorical data. The unpaired *t* test was used for continuous data. We used univariate and multivariate logistic regression models to examine the association between overweight and the prevalence of FA. The models included a crude model, adjusted for age (continuous variable) or age and asthma. All statistical tests were two-tailed. *p* values less than 0.05 were regarded as significant. All analyses were performed using IBM SPSS Statistics version 19.0 for Windows.

### Ethics approval

This study was approved by the Ethics Committee of Kanazawa University and adhered to the ethical guidelines of the Declaration of Helsinki. We obtained informed consent for study participation from each of the children and from a parent or guardian.

## Results

Table 1 shows the characteristics of the participants. In total, 1772 children (878 boys and 894 girls) were analyzed. The prevalence of overweight in boys (19.9%) was significantly higher than in girls (12.9%) (*p* < 0.001).

**Table 1:** General characteristics of participants

	Boys		Girls		*p* value^a^
	(*n* = 878)		(*n* = 894)		
Age groups (*n*, %)					
3–6 years	191	(21.8)	207	(23.2)	0.838
6–9 years	216	(24.6)	223	(24.9)	
9–12 years	232	(26.4)	222	(24.8)	
12–15 years	239	(27.2)	242	(27.1)	
Height (cm, mean, SD)					
3–6 years	107.1	(7.2)	104.8	(7.2)	**0.001**
6–9 years	125.2	(7.7)	124.2	(7.8)	0.179
9–12 years	144.5	(9.7)	143.7	(8.2)	0.321
12–15 years	162.5	(9.0)	155.3	(5.1)	**< 0.001**
Weight (kg, mean, SD)					
3–6 years	18.0	(3.3)	17.1	(3.1)	**0.004**
6–9 years	26.6	(6.3)	25.2	(5.4)	**0.016**
9–12 years	40.0	(10.5)	37.7	(7.6)	**0.008**
12–15 years	53.5	(12.7)	47.7	(7.7)	**< 0.001**
BMI (kg/m^2^, mean, SD)					
3–6 years	15.6	(1.6)	15.5	(1.6)	0.453
6–9 years	16.8	(2.8)	16.2	(2.2)	**0.015**
9–12 years	18.9	(3.4)	18.1	(2.7)	**0.008**
12–15 years	20.1	(3.8)	19.7	(2.8)	0.261
Weight groups (*n*, %)					
Overweight	175	(19.9)	115	(12.9)	**< 0.001**
Non-overweight	703	(80.1)	779	(87.1)	
Prevalence of food allergy (*n*, %)					
With food allergy	65	(7.4)	56	(6.3)	0.342
Without food allergy	813	(92.6)	838	(93.7)	
History of asthma (*n*, %)					
With asthma	200	(22.8)	125	(14.0)	**< 0.001**
Without asthma	678	(77.2)	769	(86.0)	

*SD* standard deviation, *BMI* body mass index

^a^Chi-square test for categorical variables and unpaired t-test for continuous variables

There was no significant difference between the prevalence of FA in boys (7.4%) and girls (6.3%). When we observed the prevalence of FA in each age group (Table 2), the boys in the 6–9 age group showed a significantly higher prevalence of FA (10.6%) in comparison with girls (4.5%) (*p* = 0.014). In contrast, girls in the 12–15 age group showed a significantly higher prevalence of FA (7.9%) than boys (2.5%) (*p* = 0.008).

**Table 2:** The differences in the prevalence of food allergy between gender by age groups

		With food allergy		Without food allergy		*p* value^a^
		(*n* = 121)		(*n* = 1651)		
Age groups		*n*	(%)	*n*	(%)	
3–6 years	Boys	21	(11.0)	170	(89.0)	0.136
	Girls	14	(6.8)	193	(93.2)	
6–9 years	Boys	23	(10.6)	193	(89.4)	**0.014**
	Girls	10	(4.5)	213	(95.5)	
9–12 years	Boys	15	(6.5)	217	(93.5)	0.787
	Girls	13	(5.9)	209	(94.1)	
12–15 years	Boys	6	(2.5)	233	(97.5)	**0.008**
	Girls	19	(7.9)	223	(92.1)	

^a^Chi-square test for categorical variables

Table 3 displays the differences in the prevalence of FA between overweight and non-overweight children by gender. In girls, the prevalence of FA in overweight (11.3%) was significantly higher than in non-overweight (5.5%) (*p* = 0.017). However, a significant relationship was not observed in boys. We examined the differences in the prevalence of FA between overweight and non-overweight children by gender and age groups (Table 4). In boys, there were no significant differences in all age groups. However, in the girls’ 12–15 age group, overweight showed a significantly higher prevalence of FA (26.1%) than non-overweight (5.9%) (*p* = 0.005). Prior to adjusting for potential confounders, a positive association between overweight and FA was observed (odds ratio; OR = 2.18, 95% confidence interval; 95% CI = 1.13–4.20). After adjusting for age as well as age and asthma, the positive relationship still remained with OR (95% CI) of 2.19 (1.14–4.22) and 1.99 (1.01–3.89), respectively (Table 5).

**Table 3:** The difference in the prevalence of food allergy between overweight and non-overweight by gender

	With food allergy		Without food allergy		*p* value^a^
	(*n* = 121)		(*n* = 1651)		
Boys	*n*	(%)	*n*	(%)	
Overweight	13	(7.4)	162	(92.6)	0.989
Non-overweight	52	(7.4)	651	(92.6)	
Girls					
Overweight	13	(11.3)	102	(88.7)	**0.017**
Non-overweight	43	(5.5)	736	(94.5)	

^a^Chi-square test for categorical variables

**Table 4:** The difference in the prevalence of food allergy between overweight and non-overweight by gender and age groups

		With food allergy		Without food allergy		*p* value^a^
		(*n* = 121)		(*n* = 1651)		
Boys		*n*	(%	*n*	%	
3–6 years	Overweight	3	(18.8)	13	(81.3)	0.393
	Non-overweight	18	(10.3)	157	(89.7)	
6–9 years	Overweight	7	(14.6)	41	(85.4)	0.316
	Non-overweight	16	(9.5)	152	(90.5)	
9–12 years	Overweight	3	(4.5)	64	(95.5)	0.563
	Non-overweight	12	(7.3)	153	(92.7)	
12–15 years	Overweight	0	(0.0)	44	(100.0)	0.596
	Non-overweight	6	(3.1)	189	(96.9)	
Girls						
3–6 years	Overweight	3	(14.3)	18	(85.7)	0.157
	Non-overweight	11	(5.9)	175	(94.1)	
6–9 years	Overweight	2	(6.7)	28	(93.3)	0.628
	Non-overweight	8	(4.1)	185	(95.9)	
9–12 years	Overweight	2	(4.9)	39	(95.1)	1.000
	Non-overweight	11	(6.1)	170	(93.9)	
12–15 years	Overweight	6	(26.1)	17	(73.9)	**0.005**
	Non-overweight	13	(5.9)	206	(94.1)	

^a^Chi-square test or Fisher’s exact test for categorical variables

**Table 5:** The association between overweight and food allergy using multiple logistic regression analysis

	OR	Unadjusted	*p* value	OR	Adjusted for age		Adjusted for age and asthma		
		95%CI			95%CI	*p* value	OR	95%CI	*p* value
Boys									
Overweight	1.00	0.53–1.89	0.989	1.19	0.63–2.27	0.593	1.13	0.58–2.19	0.718
Non-overweight	1	(reference)		1	(reference)		1	(reference)	
Girls									
Overweight	2.18	1.13–4.20	**0.019**	2.19	1.14–4.22	**0.019**	1.99	1.01–3.89	**0.046**
Non-overweight	1	(reference)		1	(reference)		1	(reference)	

*OR* odds ratio, *CI* confidence interval

## Discussion

The purpose of the present study was to examine the association between overweight and the prevalence of FA among Japanese children. We found that the prevalence of FA was significantly higher in overweight than non-overweight girls in the 12–15 age group; however, no significant difference was found in boys. Furthermore, overweight was significantly associated with FA after adjustment for age and asthma in girls. Our results showed that being overweight was significantly associated with a higher prevalence of FA in girls, but not in boys.

In the present study, the prevalence of food allergy is 8.1% (boys 8.8%, girls 6.8%) in 3–6-year-old children. This value is comparable with that of nationwide Japanese survey [5]. In the present study, the prevalence of food allergy is 6.3% (boys 6.4%, girls 6.1%) in 7–15-year-old children. The value is higher than that of the nationwide study [5]; however, our study did not include high school children.

We observed that the prevalence of FA in boys in the 6–9 age group was significantly higher than in girls, although, in the 12–15 age group, girls had a significantly higher prevalence of FA than boys. This reversed relationship between gender and age was similar to a previous study showing that the prevalence of FA and asthma in boys was higher than in girls before puberty and that the prevalence of FA and asthma in girls was higher than boys after puberty [17–19]. A possible explanation for the relationship between gender and FA is that estrogens enhance humoral immunity and antibody synthesis, while androgens and progesterone suppress immunity and inflammation [20]. Increased estrogen with age in girls may result in the increased prevalence of FA with age.

In this study, we examined the relationship between overweight and the prevalence of FA. A positive relationship between overweight and the prevalence of FA in girls was found in the present study. A previous study in the USA exploring the relationship between obesity, serum IgE, and allergic symptoms concluded that obesity may be a contributor to the increased prevalence of allergic disease in children, particularly FA [21].

On the other hand, a study of Vietnamese children found that there was no significant association between overweight and FA [22]. However, in this study, 9.0% of participants were underweight, while 0.6% were overweight. It is likely that this low prevalence of overweight is one reason that there was no significant relationship between overweight and FA in Vietnamese children.

Our study showed a significant association between overweight and the prevalence of FA only in girls. A possible explanation for the relationship between overweight, FA, and gender, in addition to the influence of sex hormones, is that overweight also may affect the development of allergies [23]. Such an association may be related to the chronic low-grade inflammation that accompanies obesity [24, 25]. Vieira et al. reported that the frequency of specific positive IgE levels in obese women is threefold higher than that found in non-obese women [26]. Visness et al. showed that C-reactive protein levels were associated with food sensitization. C-reactive protein is a marker for systemic inflammation and is often very high in overweight individuals. They suggested that systemic inflammation may play a role in the development of allergic diseases [21]. Besides inflammation, the effect of microbiota could also be involved in FA. Microbiota have been reported to influence both the immune system and obesity [27, 28].

Since this study is a cross-sectional study, a major limitation is that we could not provide a causal relationship. Further prospective studies are necessary to find a causal relationship between overweight and FA. Another limitation is that diagnosis of allergic diseases was self-reported, which would require objective measurement methods such as a skin prick test or an oral food challenge test. However, we can provide convincing data with good reliability, because this study was performed with a very high response rate to the questionnaires. The number of the participants seems to be too small to conduct stratified analysis shown in Tables 2 or 4. Further study using larger samples are needed to confirm the result in the present study.

## Conclusions

Our results showed that being overweight was significantly associated with a higher prevalence of FA in girls, but not in boys. Preventing or decreasing the amount of overweight might lead to a decrease in the prevalence of FA in girls. Further prospective studies are necessary to find a causal relationship between overweight and FA.

## Data Availability

The datasets generated during and/or analyzed during the current study are not publicly available due to specific restriction from the ethics committee, but are available from the corresponding author on reasonable request.

## Abbreviations

BMI: Body mass index
CI: Confidence interval
FA: Food allergy
ISAAC: International Study of Asthma and Allergies in Childhood
OR: Odds ratio
SD: Standard deviation
